# Editorial: How does brain stimulation work? Neuroversion and other putative mechanisms of action

**DOI:** 10.3389/fpsyt.2024.1488846

**Published:** 2024-09-26

**Authors:** Sujita Kumar Kar, Amílcar Silva-dos-Santos, Mikhail A. Lebedev, Zhi-De Deng

**Affiliations:** ^1^ Department of Psychiatry, King George’s Medical University, Lucknow, India; ^2^ Department of Neuroscience, Hospital Cuf Tejo, Lisboa, Portugal; ^3^ Department of Psychiatry, Hospital Dr. José de Almeida, Cascais, Portugal; ^4^ Department of Mathematical Analysis, Faculty of Mechanics and Mathematics, Lomonosov Moscow State University, Moscow, Russia; ^5^ Laboratory of Neurotechnologies, Sechenov Institute of Evolutionary Physiology and Biochemistry of the Russian Academy of Sciences, Saint-Petersburg, Russia; ^6^ National Institute of Mental Health, National Institutes of Health, Bethesda, MD, United States

**Keywords:** neuromodulation, brain stimulation, electroconvulsive therapy (ECT), transcranial magnetic stimulation (TMS), transcranial direct current stimulation (tDCS), neuroversion

Brain stimulation is an evolving treatment modality with significant utility in the management of various psychiatric and neurological disorders. Historically, electroconvulsive therapy (ECT) is the oldest brain stimulation technique in psychiatry and served as the only available neuromodulation technique for several decades. Over the past 50 years, however, advances in the field have introduced several other brain stimulation techniques, including deep brain stimulation (DBS), repetitive transcranial magnetic stimulation (rTMS), transcranial direct current stimulation (tDCS), vagus nerve stimulation (VNS), and magnetic seizure therapy (MST). These techniques employ either electrical or electromagnetic stimuli to trigger neurochemical, electrophysiological, and neuroimmune changes in the brain that in turn lead to therapeutic effects (1–4). With the increasing use of these brain stimulation techniques and expanding research efforts to understand their mechanisms of action, our knowledge of how these treatments work continues to grow.

The Research Topic “How Does Brain Stimulation Work? Neuroversion and Other Putative Mechanisms of Action” brings together research on various approaches to brain stimulation that is expected to enrich the understanding of researchers and clinicians to stimulate further research and influence practice. This Research Topic includes 10 articles authored by 76 authors covering a broad range of brain stimulation techniques, including ECT, tDCS, rTMS, DBS, and theta burst stimulation (TBS) in various neuropsychiatric disorders. Of the 10 articles, four are case reports and the remaining six are original research, including two clinical trials.

In a case report, Katzell et al. discussed the use of ECT in a patient with refractory status epilepticus on VNS. This patient had a history of developmental delay, and traumatic brain injury with subsequent sequelae of hygroma, which resulted in the development of progressive aphasia, status epilepticus, and a deteriorating sensory level. The patient received right anterior, and left temporal (RALT) ECT followed by sessions of bitemporal ECT, with no improvement in status epilepticus. In another case report, Katzell et al. reported on the beneficial role of ECT in the treatment of Neuroleptic Malignant Syndrome (NMS). Another prospective study investigated a potential therapeutic mechanism of ECT (Erchinger et al.). The levels of brain N-acetyl aspartate were temporarily reduced following ECT in patients with moderate to severe depression and normalized within six months following ECT. Deep brain stimulation was used in the management of a patient with intractable obsessive-compulsive disorder (OCD) in a case study by Beydler et al. In this case study a 58-year-old woman with childhood-onset OCD who did not respond to trials of serotonergic medications, psychotherapy, or even ECT was treated with DBS targeting the anterior limb of the internal capsule and nucleus accumbens. The patient showed significant improvement following DBS.

Another case report by Beydler et al., discussed the role of high-frequency rTMS in the management of a mixed affective state. The patient was a 68 year-old woman, who did not respond to adequate trials of multiple mood stabilizers and antipsychotic medications. She received nine sessions of high-frequency rTMS over the left dorsolateral prefrontal cortex at 120% of motor threshold (MT), with significant improvement in the affective symptoms. Senczyszyn et al., conducted a pilot randomized controlled trial by using rTMS in older adults with mild cognitive impairment. Patients with mild cognitive impairment receiving high-frequency rTMS alone or in combination with computer-based cognitive rehabilitation showed significant improvement in their overall cognitive function. Zhang et al. conducted a randomized crossover study involving 20 young healthy adults who received single sessions of intermittent theta burst stimulation (iTBS) at serially increasing MTs (50%, 70%, or 100%) on separate occasions with serial monitoring of brain physiology by using functional near-infrared spectroscopy (fNIRS). A U-shaped response (non-linear) was found between the change in MT and prefrontal hemodynamic changes following iTBS intervention. Quinn et al. also evaluated the role of iTBS delivered in an accelerated fashion over the right dorsolateral prefrontal cortex in late-life depression. A positive correlation was found between the electric field over the ventrolateral prefrontal cortex and the antidepressant effect, whereas a negative correlation was found between the posterior dorsolateral prefrontal cortex and the antidepressant effect.

Isik et al. explored the combination of tDCS with auditory stimulation to test the entrainment of frontal alpha activity during interventions under general anesthesia. Although the intervention was found to be safe and feasible, it was not found to increase alpha power. Lee et al. used tDCS in the treatment of bipolar depression as an adjuvant treatment modality in the home setting. This randomized controlled trial did not find any superiority of active tDCS over sham tDCS in the management of bipolar depression.

Overall, this Research Topic highlights the diverse applications of brain stimulation techniques and the emerging evidence supporting their benefits. As the research in this field advances, these findings will help refine clinical practice and stimulate further investigation into the mechanisms underlying brain stimulation therapies.
